# Draft Genome Sequence of *Bacillus plakortidis* P203^T^ (DSM 19153), an Alkali- and Salt-Tolerant Marine Bacterium

**DOI:** 10.1128/genomeA.01690-15

**Published:** 2016-02-04

**Authors:** Jie-ping Wang, Bo Liu, Guo-hong Liu, Ci-bin Ge, Rong-feng Xiao, Xue-fang Zheng, Huai Shi

**Affiliations:** Agricultural Bio-Resources Research Institute, Fujian Academy of Agricultural Sciences, Fuzhou, Fujian, China

## Abstract

*Bacillus plakortidis* P203^T^ is a Gram-positive, spore-forming, and alkali- and salt-tolerant marine bacterium. Here, we report the 3.97-Mb draft genome sequence of *B. plakortidis* P203^T^, which will promote its fundamental research and provide useful information for genomic taxonomy and phylogenomics of *Bacillus*-like bacteria.

## GENOME ANNOUNCEMENT

The type strain P203^T^ (=CIP 107762^T^) of *Bacillus plakortidis* was isolated from material from the sponge *Plakortis simplex* and identified as a novel halo- and alkali-tolerant species of the genus *Bacillus* (1). Up to now, no additional information of *B. plakortidis* has be obtained, except that it can be found in an extremely alkaline bauxite residue site of an alumina industrial plant (2). Because of no available genomic information of *B. plakortidis*, its type strain P203^T^ was selected as one of the research objects in our genome sequencing project for genomic taxonomy and phylogenomics of *Bacillus*-like bacteria. Here, we present the first draft genome sequence of *B. plakortidis*.

The genome sequence of *B. plakortidis* P203^T^ was obtained by paired-end sequencing on the Illumina HiSeq 2500 system. One DNA library with an insert size of 456 bp was constructed and sequenced. After filtering of the 0.55-Gb raw data, the 0.53-Gb clean data were obtained, providing approximately 134-fold coverage. The reads were assembled via SOAPdenovo software version 1.05 (3), using a key parameter K setting at 76. Through the data assembly, 44 scaffolds with a total length of 3,967,762 bp were obtained, and the scaffold *N*_50_ was 227,279 bp. The average length of the scaffolds was 90,176 bp, and the longest and shortest scaffolds were 535,841 bp and 673 bp, respectively. A total of 96.36% clean reads could be aligned back to the genome, which covered 98.19% f the sequence.

The annotation of the genome was performed using the NCBI Prokaryotic Genomes Automatic Annotation Pipeline (PGAAP) (http://www.ncbi.nlm.nih.gov/genome/annotation_prok) utilizing GeneMark, Glimmer, and tRNAscan-SE tools (4). A total of 4,216 genes were predicted, including 4,143 coding sequences, 68 tRNAs, and 5 rRNAs. There were 2,988 and 1,816 genes assigned to the COG and KEGG databases, respectively. The average DNA G+C content was 39.79 %, agreeing with the value 41.1 mol% (1).

### Nucleotide sequence accession numbers.

This whole-genome shotgun project has been deposited at DDBJ/EMBL/GenBank under the accession number LJJD00000000. The version described in this paper is the first version, LJJD01000000.
